# Impact of the 2011 heat wave on mortality and emergency department visits in Houston, Texas

**DOI:** 10.1186/1476-069X-14-11

**Published:** 2015-01-27

**Authors:** Kai Zhang, Tsun-Hsuan Chen, Charles E Begley

**Affiliations:** Department of Epidemiology, Human Genetics and Environmental Sciences, University of Texas School of Public Health, Houston, Texas 77030 USA; Department of Management, Policy and Community Health, University of Texas School of Public Health, Houston, Texas USA

**Keywords:** Emergency department visits, Heat wave, Mortality, Ozone, Temperature

## Abstract

**Background:**

Heat waves have been linked to increased risk of mortality and morbidity, and are projected to increase in frequency and intensity in a changing climate. Houston and other areas in Texas experienced an exceptional heat wave in the summer of 2011 producing the hottest August on record. This study aims to assess the health-related impact of this heat wave.

**Methods:**

Distributed lag models were used to estimate associations between the 2011 heat wave and all-cause mortality and emergency department (ED) visits from May 1 through September 30 for the five-year period 2007–2011. The 2011 heat wave is defined as a continuous period from August 2 through 30, 2011 according to the heat advisories issued by the local National Weather Service office, and is included in the models as a dummy variable. We compared the estimated excess risk among the models with and without adjustment of continuous temperature and ozone.

**Results:**

The 2011 heat wave in Houston was associated with a 3.6% excess risk in ED visits (95% CI: 0.6%, 6.6%) and 0.6% increase in mortality risk (95% CI: -5.5%, 7.1%). The elderly over 65 years of age were at the greatest risk in ED visits. These patterns are consistent across different heat-wave definitions, and results are similar when adjusting for continuous temperature and ozone.

**Conclusions:**

The 2011 heat wave in Houston had a substantial impact on ED visits and no significant impact on mortality. Our findings provide insights into local heat-wave and health preparations and interventions.

**Electronic supplementary material:**

The online version of this article (doi:10.1186/1476-069X-14-11) contains supplementary material, which is available to authorized users.

## Background

Heat waves have been shown to be associated with excess risks of mortality and morbidity [1–3]. For examples, the 1995 Chicago heat wave was estimated to result in 692 excess deaths during the heat wave [4] and 1072 excess hospital admissions [5]; The July 2006 California heat wave was estimated to have caused 215 to 505 excess deaths in nine counties of California compared to July 1999–2005 [2]; 16,166 excess emergency department (ED) visits and 1,182 excess hospital admissions statewide compared to a reference period in 2006 [1]; The 2003 summer heat in France resulted in 14,800 excess deaths [3].

During the summer 2011, an exceptional heat wave affected Texas, Oklahoma, and the surrounding portions of Arkansas, Kansas, Louisiana and New Mexico [6]. The average temperature from June to August in Texas was 30.4°C, which broke all previous single-month records and was 2.9°C higher than the long-term climatological average [6]. The 2011 heat wave is more severe than previous extreme heat waves on record in Texas which occurred in 1934, 1980 and 1998, and its extreme magnitude is much greater than the Texas summer temperature records since 1895 [6]. In Houston, many historical temperature records were broken. For examples, the 2011 summer (overall average temperature: 31.1°C; and average daily maximum temperature: 37.1°C) was warmer than any previous summer since 1889; the mean temperature of August (32.4°C) was the highest among the temperature records of all previous months; and all-time highest temperature (42.8°C) occurred on August 27th [7]. Additionally, 30 out 31 days in August in Houston had daily maximum temperature above 100 degree Fahrenheit (37.8°C), and this led to the most consecutive days with issued heat advisories (August 2 to 30) by National Weather Service (Wood, L, personal communication, January 30, 2013). The effects of land use on temperatures during the 2011 heat wave in Houston has been examined across a spectrum of land use variables using land use regression and quantile regression, and distance to the coastline, built environment, open water and vegetation have been found to be statistically significantly associated with temperature measurements [8]. However, to the best of our knowledge, the health effects of the 2011 heat wave in Texas have not been reported.

This paper aims to examine impact of this extreme event on mortality and ED visits in Houston, the fourth largest city in US [9]. We applied a time series analysis approach to determine whether excess deaths and ED visits occurred during the 2011 heat wave by modeling daily counts of deaths and ED visits as a function of the 2011 heat wave using different heat-wave definitions.

## Methods

### Study location

This study was conducted in Harris County, Texas, where the city of Houston is located. Harris County was the third most populous county in the US, and had an estimated population of 4.1 million in 2010 [10]. The Harris County population included 56.6% white, 18.9% black or African American, 6.2% Asian and some other races, and 40.8% of residents were Hispanic [11]. Nearly 8.1% of Harris County’s population was over 65 years of age [11] and 17.9% had incomes below the federal poverty level [12]. Houston has a humid subtropical climate because it is close to the Gulf of Mexico. The summer average temperature during 1981–2010 was 28.78°C (http://www.ncdc.noaa.gov/cdo-web/datatools/normals). This study was approved by the Committee for the Protection of Human Subjects of the University of Texas Health Science Center at Houston (HSC-SPH-13-0085 and HSC-SPH-14-0240).

### Mortality data

This study used all-cause mortality of residents living within Harris County from 2007 to 2011. Original death records were obtained from the Texas Department of State Health Services and were aggregated on a daily basis at the county level. The International Classification of Disease (ICD) Tenth Revision (ICD-10) was used for the diagnosis of diseases, and the primary causes of death were restricted to internal causes (ICD-10: below S) or external causes due to extreme heat (ICD-10: X30 and T67). Data for mortality by individuals living within Harris County were included in this study.

### ED visit data

This study utilized all-cause ED visit data in Harris County from 2007 through 2011 originally collected in the Harris County Hospital Emergency Department Use Study [13]. This study surveyed ED visits at all hospital-based emergency rooms in general hospitals that accepted 911 ambulance deliveries. The number of participating hospitals varied with years as the study was voluntary, e.g., only five hospitals were included in 2002 while 25 hospitals were included in 2011. This paper used the ED data from 20 hospitals which continuously participated in the ED use study during 2007–2011. Diagnoses were coded using ICD Ninth Revision (ICD-9), and this research was limited to all-cause ED data if ICD-9 codes were less than 800 (internal causes) or equal to 992 or E900 (due to extreme heat). The dates of admission for ED visits were used to match patients’ ED visits to temperature exposures. Data for ED visits by individuals residing within Harris County were included in this study.

### Weather and air pollution data

Hourly weather data at the weather station located in the George Bush International Airport during 2007–2011 were downloaded from the National Climate Data Center through the Integrated Surface Database (http://www.ncdc.noaa.gov/isd). We excluded those days with more than 25% of hourly values were missing and then calculated daily maximum, mean, minimum temperatures and apparent temperatures, a composite index of both temperature and relative humidity [14].

Ozone (O_3_) measurements in Harris County were retrieved from the Texas Air Monitoring Information System database (http://www.tceq.state.tx.us/goto/tamis). We restricted ozone data to those days on which 75% or more of hourly ozone measurements were available and then derived county-wide daily mean O_3_ concentrations by taking the averages across all available monitors (17 monitors during 2007–2010 and 20 monitors in 2011). Most of ozone monitors are located in the eastern and southern of Harris County.

### Statistical analysis

We merged mortality data, ED visit data, weather observations and air pollution data by date. How heat waves are modeled has differed among studies. Some studies include a dummy variable for heat waves in models [15] while others adjust for continuous temperature besides a dummy variable [16]. Because there is no consensus on the definition of heat wave, we first defined the 2011 heat wave in Houston as those days (August 2–30, 2011) when heat advisories were issued by the National Weather Service Houston/Galveston office. This definition includes 29 days, which is much longer than typical heat waves around a few days or one week. Thus, we explored other three percentile-based definitions which were periods of 2 or more consecutive days in 2011 August with daily mean temperatures above the 95th, 97th, or 99th percentiles of the daily mean temperature distribution in 2011.

We first conducted a crude analysis comparing deaths and ED visits during the 2011 heat wave period to reference periods in 2007 through 2010. Then, we applied distributed lag non-linear models (DLNMs) to quantify the excess risks of mortality and ED visits in the heat wave period compared to warm months (May 1st to September 30th) from 2007 through 2011. DLNMs capture non-linear exposure-response dependencies and delayed effects simultaneously, and have been used in quantifying the effects of heat and heat waves [17]. In this study, we used a dummy variable to represent the 2011 heat wave and then estimated the cumulative effects of the 2011 heat wave by considering its lag effects using DLNMs. The maximum number of lags was set at 7 to allow us to capture delayed effects and the “harvesting” phenomenon (excess deaths occur during a heat wave whereas a decrease of death counts is observed within 7 days following this heat wave), which is consistent with Tong et al. [15]. The lag dimension was modeled using a natural spline function with 5 degrees of freedom. We assumed daily counts of deaths and ED visits following an over-dispersed Poisson distribution and modeled them as:
1

where *Y*_*t*_ is the number of deaths or ED visits on day *t*; α is the intercept; *HW*_*t* - *q*_ is a dummy variable for the 2011 heat wave (1 if day t was classified as part of the 2011 heat wave, 0 otherwise) and represents the heat-wave exposure delayed over time for *q* days, and *β* is the vector of the coefficients for the heat-wave variables; *DOW*_*t*_ is a set of dummy variables for day of the week, and *γ* is the vector of regression coefficients; *Year*_*t*_ represents a long-term trend; *DOY*_*t*_ represents day of year with 4 degrees of freedom to account for seasonality. DLNMs were fit using the “dlnm” R package (version 2.0.6) [17] in the R statistical software (R Development Core Team; http://R-project.org). Additionally, the very young and the elderly have been shown to be vulnerable to heat [1, 18], and thus we applied eq. (1) to three age groups: less than 6 years of age representing infants/toddlers/preschool kids according to the American Academy of Pediatrics definition on the very young children [19]; 6 to 65 years of age; and above 65 years of age.

### Sensitivity analysis

We conducted sensitivity analyses to evaluate whether estimated heat wave effects on mortality and ED visits varied with adjustment for continuous apparent temperature and ozone. We explored the heat-wave effects with adjustment for two-day moving averages of continuous apparent temperature on the current day and the previous day, which was modeled as a natural spline with 6 degrees of freedom. We chose apparent temperature because it reflects both temperature and relative humidity, and absolute apparent temperature has been shown to be a robust predictor of all-cause mortality in four US cities [20]. We also modeled the same-day ozone concentration with mortality and ED visits counts. In addition, we evaluated whether results are sensitive to the choice of degrees of freedom in the lag space by choosing degrees of freedom of 3 and 7 compared to 5 in our default setting. Finally, we adopted a few more heat-wave definitions by extending the minimum heat-wave duration from 2 days to 4 days or using the 95th, 97th, or 99th percentiles of the daily mean temperature distributions during 1980–2010.

## Results

Table 1 shows temperatures, and the number of deaths and ED visits during the 2011 heat wave/summer and reference periods. The average daily maximum, mean and minimum temperatures during the event (August 2–30, 2011) were 3.7, 2.5, and 1.4°C higher than their respective levels during the same days during 2007–2010. Typically, there was one more elderly death during the event compared to each of the previous years. Approximately 278 more ED visits occurred during the event, and 66% and 23% of these excess visits were contributed by people between 6 and 65 years of age and above 65 years of age, respectively. After adjusting for population difference among age groups, age-standardized rates were 8, 6 and 19 per 100,000 people for three age groups (0–6 years, 6–65 years, >65 years), respectively. During the entire summer (June 1 to August 31, 2011), the differences of the temperature metrics and ED visits between the 2011 summer and the reference periods were generally less than those differences between the 2011 heat wave period and its reference period.

**Table 1:** **Descriptive statistics for mean and range of meteorological variables, mortality and ED visits during the 2011 heat wave and summer in Harris County, Texas**

Variable’s description	Unit	2011 Heat-wave period	Reference period ^a^	Differences	2011 Summer period ^b^	Reference period ^c^	Differences
Daily minimum temperature	°C	26.3 (23.9, 27.8)	24.9 (22.2, 28.3)	1.4	25.3 (21.1, 28.3)	24.5 (17.8, 28.3)	0.9
Daily mean temperature	°C	32.3 (29.4, 34.7)	29.7 (25.0, 32.8)	2.5	30.9 (25.0, 34.7)	29.2 (24.4, 33.1)	1.7
Daily maximum temperature	°C	38.2 (34.4, 42.2)	34.5 (27.2, 38.3)	3.7	36.5 (28.9, 42.2)	34.0 (27.2, 39.4)	2.5
Daily all-cause mortality counts	Counts	52 (40, 67)	51 (32, 68)	1	52 (32, 67)	51 (32, 74)	2
Daily all-cause mortality counts among person less than 6 years old	Counts	1 (0, 4)	1 (0, 4)	0	1 (0, 4)	1 (0, 4)	0
Daily all-cause mortality counts among person between 6 and 65 years old	Counts	16 (10, 26)	16 (6, 30)	0	16 (7, 26)	17 (6, 30)	0
Daily all-cause mortality counts among person above 65 years old	Counts	35 (25, 47)	34 (22, 50)	1	35 (18, 47)	33 (18, 59)	2
Daily counts of all-cause ED visits	Counts	2064 (1795, 2406)	1786 (1476, 2110)	278	2039 (1795, 2406)	1795 (1476, 2199)	244
Daily counts of all-cause ED visits among person less than 6 years old	Counts	303 (248, 404)	271 (202, 425)	32	311 (239, 404)	284 (202, 449)	27
Daily counts of all-cause ED visits among person between 6 and 65 years old	Counts	1456 (1242, 1712)	1272 (1058, 1527)	184	1422 (1226, 1712)	1265 (1016, 1621)	157
Daily counts of all-cause ED visits among person above 65 years old	Counts	305 (236, 351)	243 (188, 302)	63	306 (236, 366)	246 (85, 329)	60

^a^2–30 August during 2007–2010; ^b^1 June to 31 August 2011; ^c^1 June to 31 August during 2007–2010.

The 2011 heat wave resulted in a 3.6% increase in ED visits (95% confidence intervals (CI): 0.6%, 6.6%) and 0.6% increase in mortality risk (95% CI: -5.5%, 7.1%) (Table 2). The heat wave increased the risk of ED visits among all age groups with the largest effect on the elderly (8.9%, 95% CI: 5.1%, 12.8%) followed by people age 6–65 year (2.8%; 95% CI: -0.1%, 5.9%) and the youngest group age 0–6 (1.4%; 95% CI: -4.6%, 7.7%). It increased the risk in mortality the most among people 6–65 years of age (3.4%; 95% CI: -7.1%, 15.1%). Using stricter percentile-based heat-wave definitions, risk of ED visits for the elderly increased to 13.1% (95% CI: 6.4%, 20.3%) (95th percentile definition) and 40.1% (95% CI: 17.0%, 67.8%) (99th percentile definition). Estimated heat-wave effects on mortality were not statistically significantly at the 0.05 level regardless of age groups and heat-wave definitions while most estimates for ED visits for all-age and the elderly groups were statistically significant for at least one of the heat-wave definitions.

**Table 2:** **Estimated excess risk (%) of the 2011 heat wave on mortality and ED visits in Harris County, Texas, by age groups and heat-wave definitions** ^**a**^

Health outcomes	Age group (years)	August 2–30, 2011	≥95th percentile	≥97th percentile	≥99th percentile
All-cause mortality	All	0.6 (-5.5, 7.1)^b^	5.0 (-5.6, 16.9)	6.0 (-8.3, 22.5)	15.8 (-15.6, 59.1)
	0 - 6	-35.6 (-61.6, 8.1)	-32.6 (-71.6, 60.2)	-56.6 (-87.0, 45.1)	-91.7 (-99.6, 75.6)
	6 -65	3.4 (-7.1, 15.1)	4.7 (-13.1, 26.1)	18.9 (-7.2, 52.2)	49.8 (-12.9, 157.7)
	>65	0.1 (-7.0, 7.8)	5.8 (-6.8, 20.1)	1.8 (-14.4, 20.9)	6.3 (-27.1, 54.8)
All-cause ED visits	All	3.6 (0.6, 6.6)	6.2 (0.9, 11.7)	7.1 (0.02, 14.7)	13.3 (-2.4, 31.5)
	0 - 6	1.4 (-4.6, 7.7)	3.3 (-7.1, 14.8)	-0.7 (-13.9, 14.6)	8.0 (-20.1, 46.1)
	6 -65	2.8 (-0.1, 5.9)	5.1 (0.02, 10.5)	6.7 (-0.3, 14.1)	9.3 (-5.7, 26.7)
	>65	8.9 (5.1, 12.8)	13.1 (6.4, 20.3)	16.9 (7.6, 27.0)	40.1 (17.0, 67.8)

^a^The 2011 heat wave was defined in four ways: August 2–30, 2011, a period with continuous heat advisories issued by the National Weather Service Houston/Galveston office; and periods of 2 or more consecutive days during August 2011 with daily mean temperature above the 95th, 97th, 99th percentiles of the daily mean temperature distribution in 2011; ^b^95% confidence intervals.

The sensitivity analysis shows that estimated excess risk attributable to the 2011 heat wave was robust with adjustment for continuous temperature and O_3_ (Table 3). For example, the point estimates of excess risk on ED visits were 3.6%, 3.1% and 3.4%, respectively, with the main model and the models with adjustment for apparent temperature and O_3_. This finding was consistent across age groups. Our sensitivity analysis on the setting of degrees of freedom in the lag space shows that our results for ED visits and mortality were robust to the choice of degrees of freedom, e.g., relative risk estimates for ED visits were 3.5% (95% CI: 0.6%, 6.6%) with 3 degrees of freedom and 3.6% (95% CI: 0.6%, 6.7%) with 7 degrees of freedom compared to 3.6% (95% CI: 0.6%, 6.6%) with 5 in our default setting. Point estimates of relative risks (see Additional file 1: Table S1) based on 4 days of duration or the 1980–2010 reference period were generally slightly smaller, but uncertainties increased somewhat, compared to those shown on Table 2.

**Table 3:** **Sensitivity analysis results of estimated excess risk (%) of the 2011 heat wave on mortality and ED visits in Harris County, Texas**

Health outcomes	Age group (years)	2011 heat-wave effects	Adjusted for apparent temperature	Adjusted for O _3_
All-cause mortality	All	0.6 (-5.5, 7.1)^a^	-0.04 (-6.3, 6.7)	0.7 (-5.4, 7.1)
	0 - 6	-35.6 (-61.6, 8.1)	-38.1( -63.6, 5.4)	-36.0 (-61.9, 7.7)
	6 -65	3.4 (-7.1, 15.1)	3.9 (-7.1, 16.1)	3.6 (-6.9, 15.3)
	>65	0.1 (-7.0, 7.8)	-0.9 (-8.3, 7.0)	0.2 (-7.0, 7.9)
All-cause ED visits	All	3.6 (0.6, 6.6)	3.1 (0.1, 6.3)	3.4 (0.5, 6.4)
	0 - 6	1.4 (-4.6, 7.7)	1.3 (-4.8, 7.8)	1.5 (-4.4, 7.7)
	6 -65	2.8 (-0.1, 5.9)	2.2 (-0.8, 5.3)	2.7 (-0.1, 5.6)
	>65	8.9 (5.1, 12.8)	9.4 (5.5, 13.6)	8.6 (4.8, 12.4)

^a^95% confidence intervals.

## Discussion

This study used consistent methods to model the effects of an exceptional heat wave on mortality and ED visits. It examined the associations between the 2011 heat wave in Houston, Texas and mortality and ED visits with a merged dataset from 2007 to 2011 using a time-series approach. The study found a significant association of the 2011 heat wave with higher ED visits but not mortality. The largest impact occurred in elderly ED visits. The findings were consistent using different heat-wave definitions and with adjustment of high temperature and O_3_.

The finding of no significant effects on mortality is contrary to previous findings from two recent exceptional heat waves in US, but is consistent with two multicity studies in the U.S. The 1995 heat wave in Chicago was associated with 692 excess deaths [4], and the 2006 California heat wave caused 215 to 505 excess deaths [2]. The estimated excess deaths from both studies were statistically significant. Possible reasons for the lack of effect in Houston include the high prevalence of air conditioning and acclimation to heat among residents. According to the 2007 American Housing Survey [21], approximately 97.9% of residences in Houston were equipped with either central air conditioning or room units while the air conditioning prevalence ratio in Chicago was 76% in 1991 [22]. Heat-related deaths have been shown to decrease with increased air conditioning prevalence [22]. Anderson and Bell [23] examined the effects of heat waves on mortality in 43 U.S. cities during 1987–2005, and found a non-significant mortality risk due to heat waves in the southern U.S. region (1.8%; 95% CI: -0.1%, 3.8%). Gasparrini and Armstrong [16] estimated heat-wave effects using a dataset including 108 U.S. cities during 1987–2000, and reported that national-average excess risks in mortality due to heat waves were not statistically significant for most heat-wave definitions. The finding may also be due to behavior change, as people in Houston might have spent more time indoors in response to heat advisories released from the local weather office during the heat wave period and earlier in the summer.

Our finding on ED visits is consistent with two previous studies in terms of the magnitude of excess risks. Knowlton et al. [1] indicated that the 2006 California heat wave was associated with 3% excess risk in all-cause ED visits (95% CI: 2%, 4%). Schaffer et al. [24] reported a 2% excess risk (95% CI: 1%, 3%) in all-cause ED visits in Sydney, Australia, as a result of the heat wave that occurred from January 30 to February, 2011. However, for ED visits, we found the 2011 heat wave in Houston had the largest impact on older people among all age groups while Knowlton et al. [1] reported the smallest excess risk on the elderly group. Previous studies have shown that older people are more vulnerable to heat [25]. In particular, a recent study examined the vulnerability of residents in Houston to heat using mortality data during 1999–2006, simulated weather data and census data and found the elderly was more sensitive to heat exposures [26].

Estimated excess risks of the 2011 heat wave were similar among the models with and without adjustment of O_3_, a continuous variable representing temperatures, with different heat wave definitions, and across age groups. This suggests that our estimates are not likely substantially confounded by O_3_ and continuous temperature. O_3_ has a slight impact on heat-wave effects, which is consistent with previous studies [14]. The minor impact of continuous temperature on estimated heat-wave effects indicates that those estimated excess risks are mainly contributed by the 2011 heat wave alone rather than high temperature. Additionally, stricter heat-wave definition based on 4 days of duration did not result in higher estimates or excess risks in mortality and ED visits partly because this definition dropped out two hottest days in August, 2011. Three percentiles of the 1980–2010 distribution of daily mean temperature had lower cut-off points compared to the 2011 distribution, and thus led to slightly lower estimates.

The findings have important implications for designing heat-wave and health warning systems and heat prevention and intervention strategies. A few metrics exist to trigger a heat-wave and health warning system, but they differ in identifying ‘dangerous heat’ [27]. Among these metrics, one metric is built on historical heat-mortality associations. This finding suggests that, if the local NWS office in Houston builds a health-based heat-wave warning system, ED visits are a better metric than mortality. Moreover, an improved understanding of risk factors modifying the effects of heat waves on ED visits (e.g., age) could help local public health and emergency departments to reduce health effects caused by heat waves through better targeted preventions and interventions.

This study has few limitations. The ED visit data used in this study do not cover all hospitals in Harris County. However, ED departments in Harris County were not required to report visits to Texas Department of State Health Service before 2014, and the data used in this study were the only available ED visits during the study period. The ED visits of participating hospitals represented a 68-76% of total ED visits to Harris County hospitals [13, 28–31]. Second, this study does not include all Texas counties because ED visit data were not available in other areas. Third, this research does not investigate the heat-wave effects on cause-specific mortality and ED visits and their spatial variation during the 2011 heat wave. Forth, exposure misclassifications occur because this study lacks personal exposures to temperature and ozone and assigns exposures using weather measurements from a single station and county-wide average ozone concentrations. This approach has been commonly used in heat-related epidemiological studies [14, 16, 23]. Exposure misclassification might be reduced to some degree by developing spatial prediction models to estimate exposures to temperature and ozone at participants’ locations. The impact of exposure misclassification errors on heat effects remains unclear. However, some studies have examined how misclassification errors influence health effects estimates in air pollution epidemiology and have shown that the impact of misclassification errors on effect estimates depend on study design, types of measurement errors (Berkson and classical errors) and others [32]. This might be true for heat related health effects, and further studies need to be conducted to quantitatively determine the impact of misclassification errors on heat effects and heat wave effects. Fifth, there has been no consensus on causes of deaths/ED visits directly and indirectly affected by heat and heat waves, and thus this study restricted mortality and ED visits to primary causes diagnosed as internal causes or ambient heat. There are some evidence suggesting potential links between heat/heat waves and mental health disorders [33], however, these associations are inconclusive. We plan to address these research questions in subsequent papers.

## Conclusion

This study is the first to examine health impact of the 2011 heat wave on mortality and ED visits. The 2011 heat wave in Houston resulted in the all-time hottest month, single day and summer ever on record. We found that the 2011 heat wave in Houston, Texas had a substantial effect on ED visits but no statistically significant effect on mortality. The excess risk in ED visits was more pronounced for the elderly than other age groups. The improved understanding of the health impact of the 2011 heat wave provides insights for local weather forecasters to design better heat-wave and health warning systems and for local government agencies and communities to design better preparation and intervention strategies to reduce adverse health effects of future heat waves.

## Electronic supplementary material

Additional file 1: Table S1: Sensitivity analysis results of estimated excess risk (%) of the 2011 heat wave using other heat-wave definitions^a^. (DOCX 14 KB)

## Abbreviations

DLNM: Distributed lag non-linear model
ED: Emergency department
ICD: International Classification of Disease.
