# Supplementary material for: Impact of the 2011 heat wave on mortality and emergency department visits in Houston, Texas
Source: Environ Health. 2015 Jan 27;14:11. doi: 10.1186/1476-069X-14-11 (PMC4417210; doi:10.1186/1476-069X-14-11)
Supplement: Supplementary file 1 — Additional file 1: Table S1: Sensitivity analysis results of estimated excess risk (%) of the 2011 heat wave using other heat-wave definitionsa. (DOCX 14 KB) [file 12940_2014_847_MOESM1_ESM.docx]

*Additional file 1: Table S1* – ***Sensitivity analysis results of estimated excess risk (%) of the 2011 heat wave using other heat-wave definitions ^a^.***

| Health outcomes | Age group (years) | ≥95th percentile  (2011)  4 days | ≥97th percentile  (2011)  4 days ^b^ | ≥95th and ≥97th (1980-2010)  2 days ^c^ | ≥99th percentile  (1980-2010)  2 days |
| --- | --- | --- | --- | --- | --- |
| All-cause mortality | All | 3.0 (-10.3, 18.3)^d^ | 2.6 (-11.8, 19.3) | 1.9 (-4.2, 8.4) | 1.3 (-5.1, 8.1) |
|  | 0-6 | -52.2 (-85.9, 62.1) | -58.6 (-89.2, 58.2) | -19.0 (-49.5, 8.4) | -24.4 (-54.7, 26.1) |
|  | 6-65 | 12.4 (-11.2, 42.4) | 13.1 (-12.7, 46.6) | 4.8 (-5.8, 29.8) | 3.7 (-7.3, 16.0) |
|  | >65 | -0.7 (-15.7, 17.1) | -1.4 (-17.7, 17.9) | 1.1 (-6.1, 8.8) | 0.8 (-6.7, 8.9) |
| All-cause ED visits | All | 4.7 (-1.9, 11.8) | 5.4 (-1.8, 13.2) | 3.8 (0.8, 6.8) | 3.8 (0.6, 7.0) |
|  | 0-6 | -5.2 (-17.4, 8.9) | -6.0 (-19.1, 9.3) | 1.6 (-4.3, 7.9) | 1.2 (-5.0, 7.8) |
|  | 6-65 | 5.4 (-1.1, 12.4) | 6.2 (-1.0, 13.8) | 3.0 (0.1, 6.0) | 3.1 (0.04, 6.3) |
|  | >65 | 10.7 (2.2, 19.8) | 12.9 (3.6, 23.1) | 9.0 (5.3, 12.9) | 9.1 (5.1, 13.2) |

^a^ Heat wave days were defined when daily mean temperature in August 2011 exceeded above the 95^th^, 97^th^ and 99^th^ percentiles of the daily mean temperature distribution in 2011 or during 1980-2010 for at least 4 or 2 consecutive days, respectively; ^b^ No heat wave days were identified when using 99^th^ percentiles for more than 4 consecutive days; ^c^ Identified heat wave days were identical when using 95^th^ and 97^th^ percentiles of 1980-2010 with 2 days of duration; ^d^ 95% confidence intervals.
